# Iron depletion by iron chelators or ferroportin inhibits HIV-1 through the induction of HIF1α, p21 and IKBα and the inhibition of CDK9 and CDK2

**DOI:** 10.1186/1742-4690-10-S1-P63

**Published:** 2013-09-19

**Authors:** Namita Kumari, Dmytro Kovalsky, Denitra Breuer, Xiaomei Niu, Sergei Nekhai

**Affiliations:** 1 Center for Sickle Cell Disease, Howard University, Washington, DC, USA; 2 ChemBio Center, National Taras Shevchenko University, Kiev, Ukraine

## Background

Intracellular iron and oxygen levels regulate HIV-1 replication by affecting several steps in the HIV-1 life cycle including transcription [1]. Low oxygen levels and reduced cellular iron are inhibitory as CDK9/cyclin T1 and CDK2/cyclin E activities are reduced and HIV-1 transcription is inhibited. The alpha subunit of hypoxia-induced factor 1 (HIF1α) is stabilized under hypoxia and in the conditions of low cellular iron. Iron depletion by iron chelators or through the expression of ferroportin, an iron export protein, inhibits CDK2 and CDK9 activities and blocks HIV-1 transcription [1]. As neither CDK2 nor CDK9 requires iron for their enzymatic activity, an apparent upstream regulation is involved. Also, induction of heme oxygenase-1 (HO-1) by hemin was shown to inhibit HIV-1 [1] although the mechanism of the inhibition was not clarified. Here we analyzed the effect of iron chelators on HIV-1 transcription and replication and also analyzed the effect of heme, a condition present in sickle cell disease that may protect against HIV-1 infection [2].

## Results

Novel iron chelators, PpY-eT and PpY-aT, efficiently inhibited HIV-1 and induced the expression of IkBα, an inhibitor of NF-kB, that was not previously reported. The chelators also induce the expression of HIF1α, increased the expression of p21, inhibited enzymatic activity of CDK2 and shifted CDK9 from the large to the small complex. HIF1α knockdown in promonocytic THP1 cells led to increased HIV-1 replication suggesting that HIF1α may restrict HIV-1. Treatment with hemin induced both HO-1 and ferroportin expression and inhibited HIV-1. Hemin treatment also induced expression of IkBα, HIF1α and p21 thus mimicking the effect of iron chelators. Peripheral blood mononuclear cells obtained from patients with sickle cell disease showed increased expression of HO-1, ferroportin, IkBα and p21 and reduced *ex-vivo* HIV-1 replication.

## Conclusions

HIV-1 transcription and replication is inhibited in low intracellular iron conditions which leads to the induction of IkBα, HIF1α and p21, resulting in inhibition of CDK2 and CDK9. The hemolytic conditions of sickle cell disease may stimulate ferroportin expression and intracellular iron reduction leading to the inhibition of HIV-1.
